# Kidney Survival Impact of Delayed Graft Function Depends on Kidney Donor Risk Index: A Single-Center Cohort Study

**DOI:** 10.3390/jcm12196397

**Published:** 2023-10-07

**Authors:** Joseba Salguero, Laura Chamorro, Enrique Gómez-Gómez, Patricia de Benito, Jose E. Robles, Juan P. Campos

**Affiliations:** 1 Urology Department, Infanta Margarita Hospital, 14940 Cabra, Spain; 2 Urology Department, Reina Sofía University Hospital, Instituto Maimónides de Investigación Biomédica de Córdoba, Universidad de Córdoba, 14004 Córdoba, Spain; laura.cc29@gmail.com (L.C.); pdebenito@hotmail.es (P.d.B.); juanp.campos.sspa@juntadeandalucia.es (J.P.C.); 3 Urology Department, University of Navarra Clinic, 31009 Pamplona, Spain; jerobles@unav.es

**Keywords:** kidney transplantation, delayed graft function, deceased donor, graft survival

## Abstract

Background: Delayed graft function (DGF) is a significant challenge in renal transplantation, particularly with deceased donors, necessitating early postoperative dialysis. The prolonged effects of medium- and long-term DGF remain uncertain, marked by contradictory graft survival outcomes. This incongruity might arise from the inherent graft resilience and regenerative capacity during transplantation. This study investigates DGF’s impact on graft survival, focusing on grafts displaying favorable (KDRI < 1) and unfavorable outcomes (KDRI ≥ 1). Methods: In this retrospective cohort study (January 2015–December 2019), we assessed kidney transplants at our center, excluding multiorgan simultaneous cases, primary non-functioning grafts, and surgical complications causing graft loss. Patients were categorized into DGF presence or absence groups. Univariate and multivariate analyses, alongside propensity score matching (PSM), were performed. Results: The study encompassed 322 deceased donor kidneys, with 83 encountering DGF. Grafts with higher KDRI indices (KDRI ≥ 1) and DGF exhibited a notably increased graft loss risk (HR: 4.17, 95% CI: 1.93–9.01). However, lower-KDRI donor grafts displayed no significant disparities between the DGF and non-DGF groups. Conclusions: Delayed graft function (DGF) development significantly contributes to graft loss in kidney transplants, particularly in grafts with KDRI ≥ 1.

## 1. Introduction

Delayed graft function (DGF) is a complication that affects the immediate postoperative period of renal transplantation, primarily in deceased donor transplantation. DGF is defined as the need for dialysis within the first seven days following renal transplantation [1].

The incidence of DGF varies depending on the type of transplanted kidney (standard or expanded criteria) and the type of donation (living donor, deceased donor with brain death, donation after circulatory death or normothermic regional perfusion), with incidences ranging from 5% to 55% [1,2,3].

DGF is influenced by multiple factors relating to both the donor and the recipient [1,2]. The deleterious effects of DGF in the immediate period are well-documented, leading to extended hospitalization, slower recipient recovery, and increased transplant costs [3,4,5].

However, the medium and long-term negative effects of DGF are not well defined, and there are contradictory findings regarding graft survival in individual studies [2]. In contrast, two meta-analyses have demonstrated that DGF constitutes a risk factor for graft loss [2,6].

The lower long-term survival in kidneys with DGF has been associated with the damage caused by ischemia reperfusion, which can lead to cell death, inflammation, and fibrosis [1,7]. Likewise, DGF has been linked to being a risk factor for graft rejection and, therefore, it can affect both patient and graft survival [2,6]. It has been theorized that delayed graft function may lead to graft fibrosis progression, but the results in this regard are contradictory [8].

This controversy regarding the impact of DGF on graft survival and functional outcomes may stem from the reserve and regenerative capacity of grafts at the time of transplantation [1], which can sometimes be assessed through preimplantation graft biopsy [9].

The aim of our study is to analyze the role of DGF in graft survival and, subsequently, examine its impact on grafts with differing survival profiles, defined as a KDRI index equal or below 1 for a more favorable profile and a KDRI index higher than 1 for a less favorable profile.

## 2. Materials and Methods

We conducted a retrospective cohort study of kidney transplantations performed at our center between 1 January 2015 and 31 December 2019, excluding patients who underwent multiorgan simultaneous transplantation, experienced primary non-functioning grafts, or had surgical complications that resulted in graft loss.

**Figure 1:**
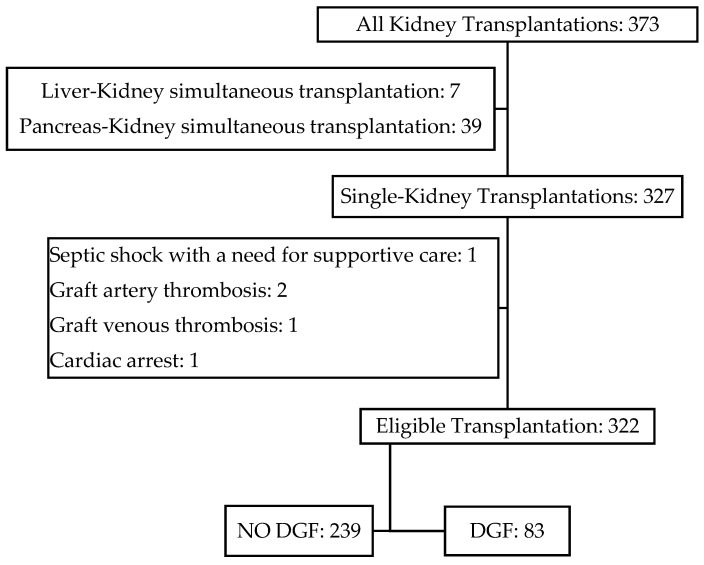
Distribution of the patients in the cohort.

The cohort was divided into two groups based on the developing of DGF (Figure 1)**,** which was defined by the need for dialysis within the first 7 days after transplantation.

The study protocol adhered to the ethical guidelines of the 1975 Declaration of Helsinki, and it received approval from the local ethics committee (Ref. N298-4365). All patients provided informed consent for kidney transplantation and data registry.

Recipient-related variables (age, sex, body mass index [BMI], history of diabetes mellitus, arterial hypertension, peripheral artery disease, cardiac disease, stroke, primary renal disease, pretransplant renal replacement therapy, time on dialysis, hyperimmunization status [panel-reactive antibody test ≥ 80%], and previous history of transplantation) (Table 1), donor-related variables (sex, age, BMI, medical history, arterial hypertension, cardiac disease, cause of death, type of donation, donor serum creatinine, and KDRI) (Table 2) and transplantation-procedure-related variables (extraction and preservation method, preimplantation graft biopsy, cold ischemic time, HLA mismatches, and number of acute rejection episodes after transplantation and follow up) (Table 3).

The rapid recovery extraction technique was executed following the declaration of death, and involved a midline laparotomy to cannulate the distal abdominal aorta, clamp the supraceliac aorta, and perfuse it with cold preservation solution, with subsequent venting through the inferior vena cava.

The normothermic regional perfusion extraction technique involves the percutaneous cannulation of the femoral artery and vein with local anesthesia before the confirmation of the patient’s death. A catheter with occlusion balloons is advanced to the thoracic aorta and inferior vena cava. After initiating life support limitation measures and verifying the patient’s demise, the occlusion balloons are deflated, and blood recirculation and oxygenation within the circuit begin, facilitating subsequent organ extraction.

Transplanted patients were administered a triple immunosuppressive regimen consisting of tacrolimus, mycophenolate mofetil, and prednisolone. Induction therapy was initiated with either an anti-interleukin-2 antibody (basiliximab) or rabbit anti-human thymocyte immunoglobulin (thymoglobulin), based on the hyperimmunized status of the patients or when the graft was obtained after circulatory death.

The preimplantation graft biopsies were acquired through wedge biopsy, preserved in formaldehyde, embedded in paraffin, and, subsequently, subjected to standard histological staining and analysis following the criteria outlined by Remuzzi et al. [10].

The qualitative variables are described as absolute and relative frequencies, the and quantitative variables as mean and standard deviation (SD) or median and interquartile range (IQR).

Chi-squared, the Fisher test, and Kruskal–Wallis tests were performed to compare the distribution of the independent variables. Kaplan–Meier and univariate and multivariate Cox regression was developed to analyze death-censored graft survival.

Propensity score matching (PSM) was performed to estimate the average marginal effect of DGF in the subgroup with KDRI ≤ 1 and in the subgroup with KDRI > 1, while accounting for potential confounding variables.

A 1:1 optimal match [11] was performed on the covariates KDRI, the time on dialysis, receptor arterial hypertension, the sex of the donor, and the presence of acute rejection. An adequate balance between the groups was defined as a standardized mean difference (SMD) for the covariates below 0.20.

The analyses were performed using SPSS (v.17.0) (IBM Inc., Chicago, IL, USA), and R (v.4.2.2) (R Foundation for Statistical Computing, Viena, Austria) was used to perform PSM analysis. A significance level of 5% (*p* < 0.05) was used to denote statistically significant differences.

## 3. Results

A total of 322 deceased donor kidneys were included in this study. Among these, 83 developed DGF. Upon analyzing recipient-related variables, patients who did not develop DGF exhibited a higher prevalence of arterial hypertension compared to those who did develop DGF (85.8% vs. 73.5%; *p* = 0.01). Additionally, a distinct distribution was observed in the prevalence of a prior history of stroke (1.6% vs. 6%; *p* = 0.05) and the mean dialysis time (mean difference: −18.52 months; *p* < 0.01) (Table 1).

Regarding the DGF graft donors, a higher proportion of males was observed (73.5% vs. 59%; *p* = 0.01), along with a greater prevalence of diabetes mellitus (20.5% vs. 11.3%; *p* = 0.03), higher basal creatinine levels (mean difference: 0.14; *p* < 0.01), and a higher Kidney Donor Risk Index (KDRI) (mean difference: 0.13; *p* = 0.01) compared to those without DGF. When donors were categorized based on the expanded criteria definition [12], the DGF group exhibited a slightly lower proportion of standard brain death donors and a slightly higher proportion of donors after cardiac death (Table 2).

The utilization of the rapid recovery extraction technique was significantly higher in the delayed graft function (DGF) group compared to the non-DGF group (24.1% vs. 13%, *p* = 0.01). However, no significant differences were observed in cold ischemia times across the various groups.

A preimplantation graft biopsy was performed when available (*n* = 119), comprising nearly 50% of the DGF grafts analyzed. Nevertheless, no significant differences were found between the groups.

During the follow-up period, the patients experiencing DGF demonstrated a prolonged postoperative hospital stay (median: 17 days vs. 11 days; *p* < 0.01) and a higher incidence of acute rejections (*p* = 0.02) (see Table 3).

**Figure 2:**
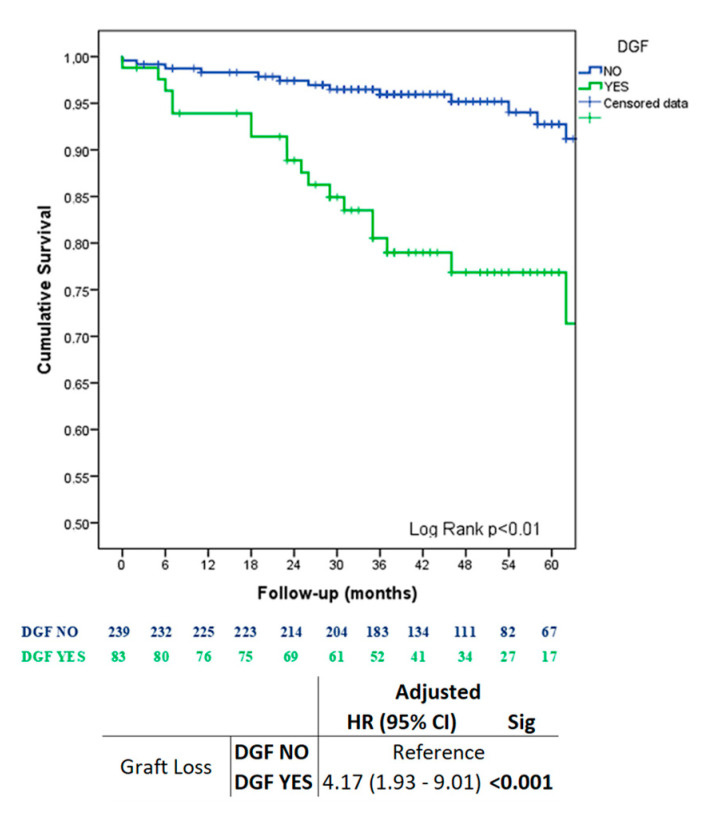
Cox analysis and Kaplan–Meier curves comparing the DGF and NO DGF groups.

The survival analysis, both univariate (HR: 4.95; 95% CI: 2.46–9.97; *p* < 0.01) and multivariate, after adjustment for the potential recipient confounding variables (HTA, ACVA, dialysis time), donor variables (sex, KDRI), and transplant-related factors (number of rejections and type of donation), confirmed that delayed graft function is an independent risk factor for graft survival (HR: 4.17; 95% CI: 1.93–9.01; *p* < 0.01) (Figure 2).

**Figure 3:**
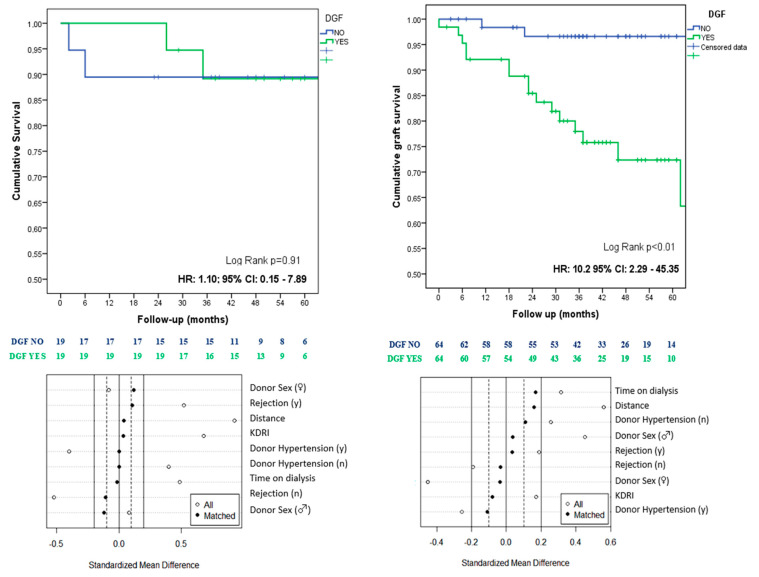
Kaplan–Meier curves comparing the DGF and NO DGF subgroups based on the KDRI index (**left** KDRI ≤ 1, **right** KDRI > 1) and standardized mean differences before and after the propensity score matching.

After PSM was performed in the subgroup with KDRI ≤ 1, 19 patients with DGF were successfully matched with 19 controls, achieving a robust adjustment. No statistically significant differences were observed in the clinical and demographic variables, as well as in the survival analysis (HR: 1.10; 95% CI: 0.15–7.89; *p* = 0.917) (Table 4) (Figure 3).

In the KDRI > 1 subgroup, PSM was also conducted, matching 64 patients with DGF to their respective controls. A satisfactory adjustment was achieved in this subgroup, as well. However, differences were noted in donor age and creatinine levels (Table 4) and in the survival analysis after adjusting for these variables (DGF: HR 10.20; 95% CI: 2.29–45.35; *p* < 0.01) (Table 4) (Figure 3).

## 4. Discussion

Delayed graft function has been identified as a crucial prognostic factor for graft survival [2,6]. It is caused by damage resulting from the ischemia-reperfusion process, which is particularly significant in recipients of grafts from expanded criteria donors and donation after circulatory death [1]. This mechanism could compromise the functionality of the nephrons within the graft.

The key characteristics that theoretically determine the development of delayed graft function (DGF) and its subsequent survival are the recovery capacity of the nephrons and their tolerance to ischemia-reperfusion damage [13].

When comparing the incidence of DGF in our cohort based on the type of donation, we found that it is similar to the reported rates in other studies for deceased donors due to brain death, but approximately 10% lower in the donation-after-circulatory-death groups [14].

These results can be attributed to the fact that, in our cohort, strategies have been implemented to reduce the cold ischemia time compared to that in the study by Kayler et al. [15], which included a cohort of 14,230 grafts with ischemia times ranging from 16.2 to 23.3 h on average.

Although individual studies have not found a direct correlation between the incidence of DGF and graft survival, meta-analyses conducted by Yarlagadda et al. [2] and Li et al. [6] revealed a higher relative risk of graft loss (HR: 1.44, 95% CI: 1.27–1.56) when combining results from multiple studies.

In our series, the development of DGF was identified as a significant risk factor for graft loss (HR: 4.17, 95% CI: 1.93–9.01) after adjusting for potential confounding variables (Figure 1), reaffirming our previous historical cohorts [16].

The theory proposing that the nature of acute damage and its reversibility may vary based on graft characteristics has attracted attention. It is suggested that standard kidneys exhibit reversible ischemic-hypoxic damage, whereas grafts with expanded criteria demonstrate irreversible chronic damage [13,17,18].

In our study, grafts with a higher KDRI index (KDRI > 1) that experienced DGF showed a significantly increased risk of graft loss (DGF: HR 10.20; 95% CI: 2.29–45.35). However, among grafts from donors with a lower KDRI, no significant differences were observed between the DGF and non-DGF groups. These findings lend support to the notion of varying graft behavior in the presence of DGF, highlighting differences in their healing capacity.

It is crucial to contextualize our findings within the framework of an experienced third-level hospital that employs a meticulously designed recipient–donor matching program.

However, it is important to approach the interpretation of all the results presented in this study with caution, as certain limitations need to be acknowledged. Notably, the retrospective nature of the study and the inclusion of patients from routine clinical practice introduce potential biases. Additionally, the relatively limited sample size might impede the broader applicability of our conclusions.

Nevertheless, our findings strongly suggest that DGF serves as a risk factor for graft loss within specific subsets of kidneys. This observation further bolsters the theory that the response of grafts to DGF can be modulated by their intrinsic characteristics. This phenomenon may offer insight into the disparate outcomes observed among different graft types and underscores the complexity of the role of DGF in kidney transplant outcomes.

It underscores the necessity for continuous research efforts to unveil the underlying mechanisms that govern graft behavior in response to DGF. Additionally, there is a need to develop non-invasive diagnostic techniques and biomarkers to accurately predict the future implications of DGF [19,20].

These findings hold substantial implications for clinical practice, as the presence of delayed graft function (DGF) results in extended hospitalization and increased transplantation costs. Thus, it is imperative to direct efforts toward strategies that effectively mitigate the long-term consequences of DGF, particularly in cases involving suboptimal grafts (KDRI > 1).

## 5. Conclusions

The development of delayed graft function (DGF) plays a pivotal role in graft loss among kidney transplant recipients, particularly in grafts with a higher Kidney Donor Risk Index (KDRI > 1).

The theory proposing distinct responses to DGF based on graft attributes is further supported by our observations, highlighting the potential for tailored interventions based on graft type.

## Figures and Tables

**Table 1:** Recipient-related characteristics.

Characteristics	All	NO DGF	DGF	Sig.
	(*n* = 322)	(*n* = 239)	(*n* = 83)	
Age (yrs), mean (SD)	55.22 (13.06)	55.49 (12.79)	54.44 (13.87)	0.74
Sex (male), *n* (%)	209 (64.9)	150 (62.8)	59 (71.1)	0.17
BMI (Kg/m^2^), mean (SD)	27.52 (5.26)	27.28 (5.23)	28.21 (5.31)	0.17
Diabetes mellitus (y), *n* (%)	73 (22.7)	49 (20.5)	24 (28.9)	0.11
Arterial hypertension (y), *n* (%)	266 (82.6)	205 (85.8)	61 (73.5)	0.01
Peripheral artery disease (y), *n* (%)	18 (5.6)	11 (4.6)	7 (8.4)	0.26
Ischemic cardiac disease (y), *n* (%)	18 (5.6)	13 (5.4)	5 (6.0)	0.78
Stroke (y), *n* (%)	9 (2.8)	4 (1.7)	5 (6.0)	0.05
Primary renal disease				0.13
Diabetes mellitus	43 (13.4)	25 (10.5)	18 (21.7)	
Hypertension	24 (7.5)	18 (7.5)	6 (7.2)	
Glomerulonephritis	60 (18.6)	45 (18.8)	15 (18.1)	
Tubulointerstitial nephritis	21 (6.5)	16 (6.7)	5 (6)	
Cystic kidney disease	53 (16.5)	44 (18.4)	9 (10.8)	
Urologic	26 (8.1)	17 (7.1)	9 (10.8)	
Unknown/missing/others	95 (29.5)	74 (31)	21 (25.3)	
Pretransplant renal replacement, *n* (%)				0.09
None	11 (3.4)	11 (4.6)	-	
Dialysis peritoneal	51 (15.8)	40 (16.7)	11 (13.3)	
Hemodialysis	260 (80.7)	188 (78.7)	72 (86.7)	
Time on dialysis (m), mean (SD)	28.74 (40.96)	23.97 (33.28)	42.49 (55.64)	<0.01
Hyperimmunized (y), *n* (%)	44 (13.7)	34 (14.2)	10 (12.0)	0.61
History of kidney transplantation (y), *n* (%)	55 (17.1)	39 (16.3)	16 (19.3)	0.53

m: months, h: hours, yrs: years, y: yes, SD: standard deviation, IQR: interquartile range, BMI: body mass index.

**Table 2:** Donor-related characteristics.

Characteristics	All	NO DGF	DGF	Sig.
	(*n* = 322)	(*n* = 239)	(*n* = 83)	
Age (yrs), mean (SD)	56.34 (14.82)	55.49 (15.70)	58.78 (11.67)	0.19
<40	38 (11.8)	33 (13.8)	5 (6)	
40–49	49 (15.2)	36 (15.1)	13 (15.7)	
50–59	86 (26.7)	61 (25.5)	25 (30.1)	
60–69	87 (27)	62 (25.9)	25 (30.1)	
≥70	62 (19.3)	47 (19.7)	15 (18.1)	
Sex (male), *n* (%)	202 (62.7)	141 (59.0)	61 (73.5)	0.01
BMI (Kg/m^2^), mean (SD)	27.68 (4.68)	27.42 (4.65)	28.42 (4.70)	0.10
Diabetes mellitus (y), *n* (%)	44 (13.7)	27 (11.3)	17 (20.5)	0.03
Arterial hypertension (y), *n* (%)	131 (40.8)	90 (37.7)	41 (49.4)	0.06
Cause of death, *n* (%)				0.39
Stroke	205 (63.7)	149 (62.3)	56 (67.5)	
Anoxia	50 (15.5)	36 (15.1)	14 (16.9)	
Head trauma	62 (19.3)	51 (21.3)	11 (13.3)	
Others	5 (1.6)	3 (1.3)	2 (2.4)	
Serum creatinine (mg/dL), median (IQR)	0.85 (0.67)	0.81 (0.68)	0.95 (0.63)	<0.01
Tipe of donation, *n* (%)				0.03
Brain death—standard donor	113 (35.1)	88 (36.8)	25 (30.1)	
Brain death—expanded criteria donor	140 (43.5)	103 (43.1)	37 (44.6)	
Donation after cardiac death	69 (24.4)	48 (20.1)	21 (25.3)	
KDRI, mean (SD)	1.26 (0.42)	1.23 (0.42)	1.36 (0.40)	0.01

m: months, yrs: years, y: yes, SD: standard deviation, IQR: interquartile range, BMI: body mass index.

**Table 3:** Transplantation-procedure-related characteristics.

Characteristics	All	NO DGF	DGF	Sig.
	(*n* = 322)	(*n* = 239)	(*n* = 83)	
Extraction and preservation method, *n* (%)				0.01
Brain death donor—cold storage	253 (78.6)	191 (79.9)	62 (74.7)	
Donation after cardiac death				
Rapid recovery—cold storage	51 (15.8)	31 (13)	20 (24.1)	
Normothermic regional perfusion—cold storage	18 (5.6)	17 (7.1)	1 (1.2)	
Preimplantation graft biopsy (score) *				0.58
≤3	32 (9.9)	20 (8.4)	12 (14.5)	
≥4	87 (27)	59 (24.7)	28 (33.7)	
Cold ischemia time (h), mean (SD)	11.67 (4.18)	11.61 (4.02)	11.85 (4.63)	0.76
HLA missmatches, median (IQR)	4 (4–5)	4 (4–5)	5 (4–5)	0.15
Number of acute rejections, median (IQR)	0 (0–1)	0 (0–0)	0 (0–1)	0.02
Hospital stay (d), median (IQR)	12 (10–15)	11 (9–13)	17 (14–20)	<0.01
Follow up (m), mean (SD)	46.07 (19.98)	47.21 (19.81)	42.77 (20.22)	0.09

h: hours, d: days, m: months, SD: standard deviation, IQR: interquartile range. * *n* = 119, NO DGF *n* = 79, DGF *n* = 40.

**Table 4:** Clinical and demographic characteristics among the PSM groups.

	KDRI ≤ 1			KDRI > 1		
Characteristics	NO DGF	DGF	Sig.	NO DGF	DGF	Sig.
	(*n* = 19)	(*n* = 19)		(*n* = 64)	(*n* = 64)	
Recipient-Related						
Age (yrs), mean (SD)	47.36 (11.2)	40.63 (15.04)	0.22	61.9 (9.59)	58.54 (10.54)	0.06
Sex (male), *n* (%)	13 (68.4)	14 (73.3)	0.72	41 (64.1)	45 (70.3)	0.45
BMI (Kg/m^2^), mean (SD)	25.08 (6.23)	27.98 (6.2)	0.14	27.68 (5.04)	28.27 (5.07)	0.58
Diabetes mellitus (y), *n* (%)	2 (10.5)	1 (5.3)	0.54	16 (25)	23 (35.9)	0.17
Arterial hypertension (y), *n* (%)	12 (63.2)	12 (63.2)	1	52 (81.3)	49 (76.6)	0.51
Peripheral artery disease (y), *n* (%)	1 (5.3)	-	1	6 (9.4)	7 (10.9)	0.77
Ischemic cardiac disease (y), *n* (%)	-	1 (5.3)	1	6 (9.4)	4 (6.3)	0.51
Stroke (y), *n* (%)	-	-	-	2 (3.1)	5 (7.8)	0.24
Pretransplant renal replacement, *n* (%)			0.6			0.21
None	-	-		3 (4.7)	-	
Dialysis peritoneal	3 (15.8)	1 (5.3)		10 (15.6)	10 (15.6)	
Hemodialysis	16 (84.2)	18 (94.7)		51 (79.7)	54 (84.4)	
Time on dialysis (m), mean (SD)	53.15 (66.91)	52.36 (47.46)	0.72	29.87 (36.57)	39.56 (57.86)	0.26
Hyperimmunized (y), *n* (%)	6 (31.6)	4 (21.1)	0.46	7 (10.9)	6 (9.4)	0.77
History of kidney transplantation (y), *n* (%)	6 (31.6)	7 (36.8)	0.73	5 (7.8)	9 (14.1)	0.25
Donor-Related						
Age (yrs), mean (SD)	44.10 (9.72)	40.63 (15.04)	0.62	66.39 (8.58)	62.46 (9.84)	0.03
Sex (male), *n* (%)	15 (78.9)	14 (73.7)	1	46 (71.9)	47 (73.4)	0.84
BMI (Kg/m^2^), mean (SD)	26.82 (5.33)	26.78 (1.99)	0.72	27.88 (4.20)	28.91 (5.16)	0.36
Diabetes mellitus (y), *n* (%)	-	-	-	15 (23.4)	17 (26.6)	0.68
Arterial hypertension (y), *n* (%)	1 (5.3)	2 (10.5)	1	33 (51.6)	39 (60.9)	0.28
Cause of death, *n* (%)			0.68			0.5
Stroke	7 (36.8)	7 (36.8)		49 (76.6)	49 (76.6)	
Anoxia	6 (31.6)	6 (31.6)		8 (12.5)	8 (12.5)	
Head trauma	5 (26.3)	6 (31.6)		7 (10.9)	5 (7.8)	
Others	1 (5.3)	-		-	2 (3.1)	
Tipe of donation			1			0.1
Brain death	13 (68.4)	13 (68.4)		52 (81.3)	49 (76.6)	
Donation after cardiac death	5 (26.3)	5 (26.3)		9 (14.1)	15 (23.4)	
Normotermic regional perfusion	1 (5.3)	1 (5.3)		3 (4.7)	-	
Serum creatinine (mg/dL), median (IQR)	0.97 (1.23)	0.73 (0.23)	0.62	0.77 (0.31)	1.02 (0.69)	0.03
KDRI, mean (SD)	0.86 (0.08)	0.86 (0.11)	0.79	1.54 (0.38)	1.51 (0.33)	0.90
Transplantation-Procedure-Related						
Cold ischemia time (h), mean (SD)	10.73 (3.91)	10.99 (5.38)	0.90	12.38 (4.05)	12.11 (4.40)	0.75
HLA mismatches, median (IQR)	3 (3–5)	4 (3–5)	0.17	5 (4–5)	5 (4–6)	0.47
Number of acute rejections, median (IQR)	0 (0–1)	0 (0–1)	0.97	0 (0–1)	0 (0–1)	0.67
Hospital stay (d), median (IQR)	11 (10–14)	14.5 (12–18.5)	<0.01	11 (9–13)	17 (14–20)	<0.01
Follow-up (m), mean (SD)	50.15 (26.50)	56.31 (16.93)	0.48	45.15 (19.70)	38.75 (19.46)	0.07

m: months, d: days, h: hours, yrs: years, y: yes, SD: standard deviation, IQR: interquartile range, BMI: body mass index.

## Data Availability

The data presented in this study are available on request from the corresponding author. The data are not publicly available due to ethical issues.
